# An Important Role for Purifying Selection in Archaeal Genome Evolution

**DOI:** 10.1128/mSystems.00112-17

**Published:** 2017-10-24

**Authors:** Zhe Lyu, Zhi-Gang Li, Fei He, Ziding Zhang

**Affiliations:** a College of Resources and Environmental Sciences, China Agricultural University, Beijing, China; b Department of Microbiology, University of Georgia, Athens, Georgia, USA; c State Key Laboratory of Agrobiotechnology, College of Biological Sciences, China Agricultural University, Beijing, China; d State Key Laboratory of Agrobiotechnology and Ministry of Agriculture Key Laboratory of Plant Pathology, Beijing, China; Institute for Genomics & Systems Biology

**Keywords:** *Archaea*, evolution, genome analysis

## Abstract

As the null hypothesis of genome evolution, population genetic theory suggests that selection strength controls genome size. Through the process of genetic drift, this theory predicts that compact genomes are maintained by strong purifying selection while complex genomes are enabled by weak purifying selection. It offers a unifying framework that explains why prokaryotic genomes are much smaller than their eukaryotic counterparts. However, recent findings suggest that bigger prokaryotic genomes appear to experience stronger purifying selection, indicating that purifying selection may not dominate prokaryotic genome evolution. Since archaeal genomes were underrepresented in those studies, generalization of the conclusions to both archaeal and bacterial genomes may not be warranted. In this study, we revisited this matter by focusing on archaeal and bacterial genomes separately. We found that bigger bacterial genomes indeed experienced stronger purifying selection, but the opposite was observed in archaeal genomes. This new finding would predict an enrichment of noncoding sequences in large archaeal genomes, which was confirmed by an analysis of coding density. In contrast, coding density remained stable regardless of bacterial genome size. In conclusion, this study suggests that purifying selection may play a more important role in archaeal genome evolution than previously hypothesized, indicating that there could be a major difference between the evolutionary regimes of *Archaea* and *Bacteria*.

**IMPORTANCE** The evolution of genome complexity is a fundamental question in biology. A hallmark of eukaryotic genome complexity is that larger genomes tend to have more noncoding sequences, which are believed to be minimal in archaeal and bacterial genomes. However, we found that archaeal genomes also possessed this eukaryotic feature while bacterial genomes did not. This could be predicted from our analysis on genetic drift, which showed a relaxation of purifying selection in larger archaeal genomes, also a eukaryotic feature. In contrast, the opposite was evident in bacterial genomes.

## Abstract (precis)

The evolution of genome complexities is a fundamental question in biology. A hallmark of eukaryotic genome complexity is that larger genomes tend to have more noncoding sequences, which are believed to be minimal in archaeal and bacterial genomes. However, we found that archaeal genomes also possessed this eukaryotic feature while bacterial genomes did not. This could be predicted from our analysis of genetic drift, which showed relaxed purifying selection in larger archaeal genomes, also a eukaryotic feature. In contrast, the opposite was evident in bacterial genomes.

## OBSERVATION

Eukaryotic genomes vary in size by orders of magnitude more than prokaryotic genomes. The genome size range is about 10^6^ to 10^11^ bp in eukaryotes, which are rich in noncoding sequences, and 10^5^ to 10^7^ bp in prokaryotes, which are streamlined and have minimal noncoding sequences (1, 2). This genome size gap is believed to be shaped primarily by nonadaptive processes based on the population genetic theory (3). That theory suggests that prokaryotes often undergo strong purifying selection owing to a generally large effective population size to maintain compact genomes (2). In contrast, eukaryotes typically have a much smaller effective population size and are subject to weak purifying selection, which enables large genomes (4). This is because all excess DNA is mutationally hazardous, and the efficiency of selection determines whether the excess DNA is removed from or fixed in the genome through the process of genetic drift (5).

The efficiency of selection can be approximately measured by the genome-wide *dN*/*dS* ratio (ratio of nonsynonymous to synonymous substitution rates) for orthologous genes shared by closely related lineages, and the stronger the purifying selection becomes, the lower the *dN*/*dS* ratio is (6). The population genetic theory predicts that bigger genomes experience weaker purifying selection or higher *dN*/*dS* ratios (2–4). However, recent findings regarding prokaryotic genomes suggest otherwise, showing that genome size is negatively correlated with the *dN*/*dS* ratio (7, 8). This only seems possible when gene gains could be slightly beneficial and that the benefits would be diluted out because of genome expansion and deletion bias (i.e., DNA loss outpaces gain), based on mathematical models (8). The benefits of gene gains thus make genome expansion possible under strong purifying selection, but the expansion stops once the benefits diminish, thus restraining the overall genome size. Indeed, deletion bias appears to be universal across the full range of cellular life forms, and its strength tends to decline when the genome expands, indicating a dynamic balance between DNA loss and DNA gain (1, 9, 10).

While bacterial species were extensively sampled in these studies, archaeal species were underrepresented (7, 8). Therefore, generalization of the mechanisms identified therein may not be warranted in *Archaea*. In this study, we concentrated on archaeal genomes to revisit previous hypotheses of genome size evolution. Bacterial genomes were also sampled and examined for comparison when necessary. We observed that the strength of purifying selection and the amount of noncoding genes were negatively and positively associated with archaeal genome size, respectively, as predicted by population genetic theory. In contrast, the opposite trend was evident in bacterial genomes.

## 

### Expansion of archaeal genomes associated with relaxed purifying selection.

In eukaryotes, relaxed purifying selection is associated with genome expansion, which is consistent with the accumulation of introns and mobile elements that are often deleterious (3).

**FIG 1:**
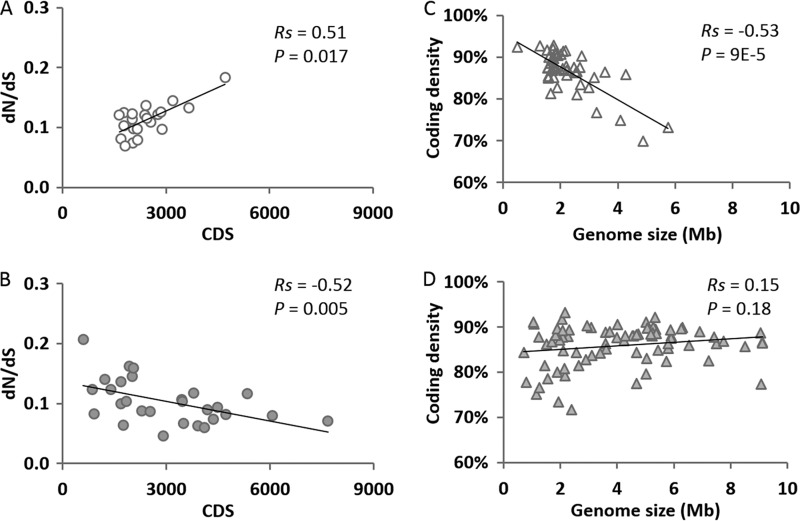
Association between genome size and the *dN*/*dS* ratio for archaeal (A; *n* = 21) and bacterial (B; *n* = 28) genome pairs and association between coding density and genome size in *Archaea* (C; *n* = 49) and *Bacteria* (D; *n* = 78). Note that the stronger the purifying selection becomes, the lower the *dN*/*dS* ratio is. Spearman rank correlation coefficients (*Rs*) and two-sided significance (*P*) values are indicated in each panel. Genome sizes are shown in megabase pairs (Mb) in panels C and D but are in protein coding sequences (CDS) in panels A and B, as only protein coding sequences were used for *dN*/*dS* ratio analysis. Regardless, the same trends were reproduced when genome size measured in base pairs instead of protein coding sequences was used.

While our bacterial data set reproduced the previous observations showing that strong purifying selection was associated with genome expansion (7, 8), our archaeal data set revealed a eukaryote-like pattern based on a genome-wide *dN*/*dS* ratio analysis (Fig. 1A and B; see Tables S1 and S2 in the supplemental material). This finding predicts an enrichment of noncoding sequences in larger archaeal genomes, as also observed in eukaryotic genomes (see below for coding density analysis).

10.1128/mSystems.00112-17.1 TABLE S1  *dN*/*dS* ratio data extracted from 21 archaeal species pairs. Download TABLE S1, DOCX file, 0.05 MB. Copyright © 2017 Lyu et al. 2017 Lyu et al. This content is distributed under the terms of the Creative Commons Attribution 4.0 International license.

10.1128/mSystems.00112-17.2 TABLE S2  *dN*/*dS* ratio data extracted from 28 bacterial species pairs. Download TABLE S2, DOCX file, 0.03 MB. Copyright © 2017 Lyu et al. 2017 Lyu et al. This content is distributed under the terms of the Creative Commons Attribution 4.0 International license.

**FIG 2:**
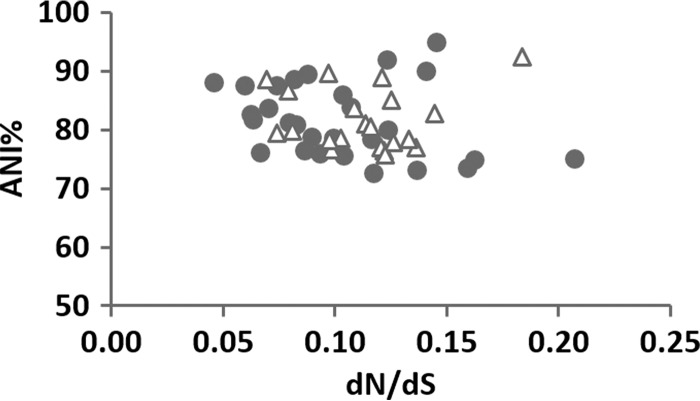
Association between the *dN*/*dS* ratio and genomic distance as measured by percent ANI. Closed circles represent bacterial pairs (*n* = 28), and open triangles represent archaeal pairs (*n* = 21).

At least a couple of observations indicated that our archaeal and bacterial data sets were comparable and likely representative. First, both archaeal and bacterial mean *dN*/*dS* ratios were between 0.05 and 0.20 (Fig. 1A and B) and a similar range has also been observed in eukaryotic lineages (11, 12). Second, most archaeal and bacterial genome pairs had an average nucleotide identity (ANI) of 75 to 95% (Fig. 2), an empirical range that delineated closely related prokaryotic species belonging to the same genus (13). This minimized the potential bias of *dN*/*dS* ratio computations caused by uneven variation in evolutionary distances between genomes (7). Nevertheless, while the bacterial samplings both here and in previous studies seem to have captured substantial taxonomic diversities of bacterial genomes, the archaeal samplings need further improvements. Sampling of archaeal genomes across more phyla will be necessary to begin to address their full genome size range. This is because the archaeal genome size narrowly ranges from 0.5 to 6 Mb compared to the 0.6- to 9-Mb range of bacterial genomes (Tables S3 and S4). Of the 29 prokaryotic phyla taxonomically established, only 2 belong to *Archaea*, where most complete archaeal genomes currently come from, and an estimated ~300 archaeal phyla still wait to be sampled (14).

10.1128/mSystems.00112-17.3 TABLE S3  Coding densities of 49 archaeal genomes. Download TABLE S3, DOCX file, 0.04 MB. Copyright © 2017 Lyu et al. 2017 Lyu et al. This content is distributed under the terms of the Creative Commons Attribution 4.0 International license.

10.1128/mSystems.00112-17.4 TABLE S4  Coding densities of 79 bacterial genomes. Download TABLE S4, DOCX file, 0.04 MB. Copyright © 2017 Lyu et al. 2017 Lyu et al. This content is distributed under the terms of the Creative Commons Attribution 4.0 International license.

### Coding density in *Archaea* shows a trend similar to that seen in eukaryotes.

Coding density or gene density, as measured by the proportion of a genome sequence that is composed of coding or gene sequence, has been shown to correlate negatively and neutrally with eukaryotic and bacterial genome sizes, respectively (7, 15, 16). This observation made in eukaryotes is consistent with a genomic structure showing that noncoding sequences, primarily spliceosomal introns and mobile genetic elements, are overrepresented in larger eukaryotic genomes (3). The observation regarding *Bacteria* is also consistent with the dynamic balance between DNA loss and DNA gain (1, 8, 9). While our bacterial data set confirmed previous observations, our archaeal data set again revealed a eukaryote-like pattern (Fig. 1C and D and Tables S3 and S4). That is to say, a strong negative correlation was observed between coding density and archaeal genome size, indicating that an insertion bias enriching noncoding sequences was also evident during the expansion of archaeal genomes. It remains elusive what category of noncoding sequences is overrepresented in larger archaeal genomes, as the nature of most archaeal noncoding sequences is poorly characterized (17). Regardless of the category, it would probably be different from that observed in eukaryotes, as spliceosomal introns have never been found in *Archaea* and the distribution of known mobile elements in archaeal genomes is similar to that in their bacterial counterparts (18). However, the presence of novel mobile elements in *Archaea* could not be ruled out. Compared to eukaryotes, one common feature is still shared by *Archaea* and *Bacteria*, both of which had a similar and narrow range of coding density, i.e., about 70 to 95% in our data set. In contrast, the eukaryotic coding density roughly ranged from 1 to 80% (15). Therefore, there could be an unknown force(s) that prevents noncoding regions in archaeal genomes from enriching to the extremely high levels observed in eukaryotes.

This study presents the first evidence that the evolutionary regimen of the complexity of archaeal genomes could be significantly different from that of their bacterial counterparts. Instead, certain key eukaryote-like evolutionary features seem to be already embedded in archaeal genomes. On the one hand, those observations seem striking, as the architecture of archaeal genomes is very similar to that of bacterial genomes (2). On the other hand, they may simply reflect the close evolutionary connections between *Archaea* and eukaryotes, since eukaryotes likely evolved within the *Archaea* (19). Although the archaeal samplings here are still limited and the similarities drawn between *Archaea* and eukaryotes here are preliminary, we believe our study serves as a sufficient reminder that it is probably no longer a safe practice to group archaeal and bacterial genomes into one category when testing evolutionary hypotheses. Rather, comparisons should be made among *Archaea*, *Bacteria*, and eukaryotes.

### Data collection and coding density calculation.

Genomic data from closely related species that belong to the same genus were downloaded from NCBI (http://www.ncbi.nlm.nih.gov/). Genera or species that were not well established, i.e., those whose taxonomic names were not formally proposed or alternative names were available, were removed before further analysis. The coding density of each genome was calculated as follows: % coding density = (DNA length of all coding sequences/total DNA length of complete genome) ×100%.

### *dN/dS* ratios and ANI analyses.

For each pair of genomes, pairs of orthologs were identified by a hybrid procedure combining Bidirectional Best Hit and BLASTclust (20). The orthologous pairs identified were aligned by ClustalW2, and *dN*/*dS* ratios were calculated by using YN00 in the PAML package (21, 22). Synonymous sites and ratios of synonymous to nonsynonymous sites considered to be saturated or unreliable (that is, a *dS* of <0.1, a *dS* of >1.6, or a *dN*/*dS* ratio of >99) were discarded before the mean *dN*/*dS* ratio and standard error of the mean of each genome pair were both calculated and reported in Tables S1 and S2. Although data filtration is a common practice to ensure better estimation of selective pressure, it also substantially reduces the number of pairwise orthologs available for downstream analyses (7, 11). This would make the data set less representative for inferring genome-wide patterns. Therefore, only genome pairs retaining pairwise orthologs accounting for no less than 5% of the pairs’ average coding capacity after all filtrations were kept for ultimate analyses. This cutoff is the starting point to represent most of the essential genes determined in prokaryotic genomes (23). ANI values were retrieved from IMG (24).

### Correlation analysis.

Spearman rank correlation (v1.0.3) was employed to obtain correlation coefficients (*Rs*) and two-sided *P* values (25).
