# Supplementary material for: An Important Role for Purifying Selection in Archaeal Genome Evolution
Source: mSystems. 2017 Oct 24;2(5):e00112-17. doi: 10.1128/mSystems.00112-17 (PMC5655593; doi:10.1128/mSystems.00112-17)
Supplement: TABLE S1 [file sys005172145st1.docx]

**Table S1** Number of orthologous genes identified from 21 archaeal species pairs*

| **Genome 1** | **Genome 2** | **No. of CDS for genomes 1/2** | **Average no. of CDS** | **No. of pair-wise orthologs** | **dN/dS**  **(Mean ± SE)** |
| --- | --- | --- | --- | --- | --- |
| *Methanobacterium paludis* SWAN-1 | *Methanobacterium congolense* Buetzberg | 2442/2373 | 2408 | 950 | 0.14 ± 0.002 |
| *Methanobrevibacter ruminantium* M1 | *Methanobrevibacter olleyae* YLM1 | 2217/1840 | 2029 | 372 | 0.07 ± 0.003 |
| *Methanobrevibacter smithii* PS | *Methanobrevibacter millerae* SM9 | 1795/2313 | 2054 | 170 | 0.10 ± 0.006 |
| *Methanocaldococcus fervens* AG86 | *Methanocaldococcus vulcanius* M7 | 1620/1765 | 1693 | 539 | 0.08 ± 0.003 |
| *Methanocaldococcus jannaschii* DSM 2661 | *Methanocaldococcus* sp. FS406-22 | 1789/1849 | 1819 | 1366 | 0.07 ± 0.002 |
| *Methanocella paludicola* SANAE | *Methanocella conradii* HZ254 | 3065/2455 | 2760 | 194 | 0.12 ± 0.006 |
| *Methanococcoides burtonii* DSM 6242 | *Methanococcoides methylutens* MM1 | 2431/2326 | 2379 | 943 | 0.12 ± 0.002 |
| *Methanococcus maripaludis* C5 | *Methanococcus vannielii* SB | 1845/1701 | 1773 | 392 | 0.10 ± 0.004 |
| *Methanoculleus marisnigri* JR1 | *Methanoculleus bourgensis* MS2 | 2506/2619 | 2563 | 1453 | 0.11 ± 0.002 |
| *Methanosarcina acetivorans* C2A | *Methanosarcina siciliae* C2J | 4721/4699 | 4710 | 2432 | 0.18 ± 0.002 |
| *Methanosarcina barkeri* Fusaro | *Methanosarcina mazei* S-6 | 3758/3548 | 3653 | 1702 | 0.13 ± 0.002 |
| *Methanosarcina thermophila* CHTI-55 | *Methanosarcina vacuolata* Z-761 | 2658/3725 | 3192 | 1890 | 0.14 ± 0.002 |
| *Methanothermobacter marburgensis* Marburg | *Methanothermobacter thermautotrophicus* CaT2 | 1757/1788 | 1773 | 1227 | 0.13 ± 0.002 |
| *Natronomonas moolapensis* 8.8.11 | *Natronomonas pharaonis* Gabara | 2847/2843 | 2845 | 163 | 0.13 ± 0.010 |
| *Pyrobaculum islandicum* DSM 4184 | *Pyrobaculum neutrophilum* V24Sta | 2014/2006 | 2010 | 658 | 0.11 ± 0.004 |
| *Pyrococcus chitonophagus* DSM 10152 | *Pyrococcus kukulkanii* NCB100 | 2153/2195 | 2174 | 1386 | 0.08 ± 0.002 |
| *Pyrococcus* sp. NA2 | *Pyrococcus furiosus* COM1 | 1980/2064 | 2022 | 427 | 0.12 ± 0.005 |
| *Staphylothermus hellenicus* P8 | *Staphylothermus marinus* F1 | 1668/1610 | 1639 | 1275 | 0.12 ± 0.002 |
| *Sulfolobus solfataricus* P2 | *Sulfolobus islandicus* HVE10/4 | 3048/2723 | 2886 | 1708 | 0.10 ± 0.002 |
| *Thermococcus eurythermalis* A501 | *Thermococcus peptonophilus* OG-1 | 2269/2050 | 2160 | 466 | 0.10 ± 0.004 |
| *Vulcanisaeta distributa* DSM 14429 | *Vulcanisaeta moutnovskia* 768-28 | 2543/2320 | 2432 | 1684 | 0.12 ± 0.002 |

*CDS: protein coding genes
