# Supplementary material for: An Important Role for Purifying Selection in Archaeal Genome Evolution
Source: mSystems. 2017 Oct 24;2(5):e00112-17. doi: 10.1128/mSystems.00112-17 (PMC5655593; doi:10.1128/mSystems.00112-17)
Supplement: TABLE S2 [file sys005172145st2.docx]

**Table S2** Number of orthologous genes identified from 28 bacterial species pairs*

| **Genome 1** | **Genome 2** | **No. of CDS for genome 1&2** | **Average no. of CDS** | **No. of pair-wise orthologs** | **dN/dS**  **(Mean ± SE)** |
| --- | --- | --- | --- | --- | --- |
| *Acinetobacter baumannii* ACICU | *Acinetobacter baylyi* ADP1 | 3667/3325 | 3496 | 369 | 0.07 ± 0.003 |
| *Actinobacillus pleuropneumoniae* L20 | *Actinobacillus succinogenes* 130Z | 2012/2079 | 2046 | 560 | 0.16 ± 0.007 |
| *Aeromonas hydrophila* ATCC 7966 | *Aeromonas salmonicida* A449 | 4122/4086 | 4104 | 2910 | 0.06 ± 0.001 |
| *Bacteroides fragilis* NCTC_9343 | *Bacteroides thetaiotaomicron* VPI-5482 | 4184/4778 | 4481 | 800 | 0.09 ± 0.003 |
| *Bartonella bacilliformis* KC583 | *Bartonella henselae* Houston-1 | 1283/1488 | 1386 | 673 | 0.12 ± 0.003 |
| *Bifidobacterium adolescentis* ATCC 15703 | *Bifidobacterium longum* F8 | 1631/1727 | 1679 | 204 | 0.10 ± 0.007 |
| *Bordetella avium* 197N | *Bordetella bronchiseptica* RB50 | 3381/4994 | 4188 | 526 | 0.09 ± 0.003 |
| *Borrelia afzelii* PKo | *Borrelia burgdorferi* B31 | 855/851 | 853 | 904 | 0.12 ± 0.004 |
| *Campylobacter concisus* 13826 | *Campylobacter curvus* 525 92 | 1929/1931 | 1930 | 873 | 0.16 ± 0.004 |
| *Candidatus* ‘Blochmannia floridanus’ | *Candidatus* ‘Blochmannia pennsylvanicus’ BPEN | 583/610 | 597 | 71 | 0.21 ± 0.016 |
| *Chlamydia muridarum* MoPn | *Chlamydia trachomatis* A HAR-13 | 904/911 | 908 | 621 | 0.08 ± 0.003 |
| *Francisella philomiragia* ATCC 25017 | *Francisella tularensis* FSC 198 | 1911/1605 | 1758 | 355 | 0.06 ± 0.003 |
| *Geobacillus kaustophilus* HTA426 | *Geobacillus thermodenitrificans* NG80-2 | 3498/3392 | 3445 | 2377 | 0.11 ± 0.002 |
| *Haemophilus ducreyi* 35000HP | *Haemophilus influenzae* R2846 | 1717/1657 | 1687 | 127 | 0.14 ± 0.022 |
| *Leuconostoc citreum* KM20 | *Leuconostoc mesenteroides* ATCC 8293 | 1702/1970 | 1836 | 159 | 0.10 ± 0.008 |
| *Listeria innocua* Clip11262 | *Listeria monocytogenes* EGD-e | 2968/2846 | 2907 | 1956 | 0.05 ± 0.001 |
| *Neisseria gonorrhoeae* FA 1090 | *Neisseria meningitidis* 053442 | 2002/2020 | 2011 | 950 | 0.15 ± 0.005 |
| *Nitrobacter hamburgensis* X14 | *Nitrobacter winogradskyi* Nb-255 | 3804/3122 | 3463 | 2016 | 0.10 ± 0.002 |
| *Pseudomonas aeruginosa* PAO1 | *Pseudomonas entomophila* L48 | 5568/5134 | 5351 | 865 | 0.12 ± 0.002 |
| *Psychrobacter arcticum* 273-4 | *Psychrobacter cryohalolentis* K5 | 2120/2467 | 2294 | 1659 | 0.09 ± 0.002 |
| *Ralstonia eutropha* H16 | *Ralstonia metallidurans* CH34 | 6206/5914 | 6060 | 1436 | 0.08 ± 0.002 |
| *Rhizobium etli* CFN 42 | *Rhizobium leguminosarum* bv viciae 3841 | 4035/4694 | 4365 | 4123 | 0.07 ± 0.001 |
| *Rickettsia canadensis* McKiel | *Rickettsia rickettsii* Sheila Smith | 1093/1345 | 1219 | 744 | 0.14 ± 0.003 |
| *Salinispora arenicola* CNS-205 | *Salinispora tropica* CNB-440 | 4917/4536 | 4727 | 3138 | 0.08 ± 0.001 |
| *Staphylococcus aureus* aureus MRSA252 | *Staphylococcus epidermidis* ATCC 12228 | 2656/2419 | 2538 | 162 | 0.09 ± 0.007 |
| *Streptomyces avermitilis* MA-4680 | *Streptomyces coelicolor* A3(2) | 7580/7769 | 7675 | 1405 | 0.07 ± 0.002 |
| *Vibrio cholerae* MJ-1236 | *Vibrio fischeri* ES114 | 3835/3761 | 3798 | 289 | 0.12 ± 0.005 |
| *Yersinia enterocolitica* 8081 | *Yersinia pestis* Angola | 3979/3837 | 3908 | 2091 | 0.06 ± 0.001 |

*CDS: protein coding genes
