# Supplementary material for: An Important Role for Purifying Selection in Archaeal Genome Evolution
Source: mSystems. 2017 Oct 24;2(5):e00112-17. doi: 10.1128/mSystems.00112-17 (PMC5655593; doi:10.1128/mSystems.00112-17)
Supplement: TABLE S3 [file sys005172145st3.docx]

**Table S3** Coding density for archaeal genomes (*n* = 49)

| Genome Name | Coding Base (bp) | Genome Size (bp) | Coding density |
| --- | --- | --- | --- |
| Aeropyrum pernix | 1.48E+06 | 1.67E+06 | 89% |
| Archaeoglobus fulgidus | 2.00E+06 | 2.18E+06 | 92% |
| Caldivirga maquilingensis IC-167 | 1.81E+06 | 2.08E+06 | 87% |
| Candidatus Korarchaeum cryptofilum OPF8 | 1.43E+06 | 1.59E+06 | 90% |
| Candidatus Methanoregula boonei 6A8 | 2.19E+06 | 2.54E+06 | 86% |
| Haloarcula marismortui ATCC 43049 | 3.67E+06 | 4.27E+06 | 86% |
| Halobacterium salinarum R1 | 2.34E+06 | 2.67E+06 | 88% |
| Halobacterium sp | 2.23E+06 | 2.57E+06 | 87% |
| Haloquadratum walsbyi | 2.50E+06 | 3.26E+06 | 77% |
| Hyperthermus butylicus | 1.36E+06 | 1.67E+06 | 81% |
| Ignicoccus hospitalis KIN4 I | 1.20E+06 | 1.30E+06 | 93% |
| Metallosphaera sedula DSM 5348 | 1.93E+06 | 2.19E+06 | 88% |
| Methanobacterium thermoautotrophicum | 1.57E+06 | 1.75E+06 | 90% |
| Methanobrevibacter smithii ATCC 35061 | 1.68E+06 | 1.85E+06 | 91% |
| Methanococcoides burtonii DSM 6242 | 2.09E+06 | 2.58E+06 | 81% |
| Methanococcus aeolicus Nankai-3 | 1.33E+06 | 1.57E+06 | 85% |
| Methanococcus jannaschii | 1.53E+06 | 1.74E+06 | 88% |
| Methanococcus maripaludis C5 | 1.56E+06 | 1.79E+06 | 87% |
| Methanococcus vannielii SB | 1.49E+06 | 1.72E+06 | 87% |
| Methanocorpusculum labreanum Z | 1.59E+06 | 1.80E+06 | 88% |
| Methanoculleus marisnigri JR1 | 2.17E+06 | 2.48E+06 | 88% |
| Methanopyrus kandleri | 1.52E+06 | 1.69E+06 | 90% |
| Methanosaeta thermophila PT | 1.56E+06 | 1.88E+06 | 83% |
| Methanosarcina acetivorans | 4.21E+06 | 5.75E+06 | 73% |
| Methanosarcina barkeri fusaro | 3.41E+06 | 4.87E+06 | 70% |
| Methanosarcina mazei | 3.07E+06 | 4.10E+06 | 75% |
| Methanosphaera stadtmanae | 1.50E+06 | 1.77E+06 | 85% |
| Methanospirillum hungatei JF-1 | 3.06E+06 | 3.54E+06 | 86% |
| Nanoarchaeum equitans | 4.54E+05 | 4.91E+05 | 92% |
| Natronomonas pharaonis | 2.48E+06 | 2.75E+06 | 90% |
| Nitrosopumilus maritimus SCM1 | 1.49E+06 | 1.65E+06 | 91% |
| Picrophilus torridus DSM 9790 | 1.42E+06 | 1.55E+06 | 92% |
| Pyrobaculum aerophilum | 1.93E+06 | 2.22E+06 | 87% |
| Pyrobaculum arsenaticum DSM 13514 | 1.84E+06 | 2.12E+06 | 87% |
| Pyrobaculum calidifontis JCM 11548 | 1.82E+06 | 2.01E+06 | 91% |
| Pyrobaculum islandicum DSM 4184 | 1.59E+06 | 1.83E+06 | 87% |
| Pyrococcus abyssi | 1.64E+06 | 1.77E+06 | 93% |
| Pyrococcus furiosus | 1.73E+06 | 1.91E+06 | 90% |
| Pyrococcus horikoshii | 1.60E+06 | 1.74E+06 | 92% |
| Staphylothermus marinus F1 | 1.38E+06 | 1.57E+06 | 88% |
| Sulfolobus acidocaldarius DSM 639 | 1.91E+06 | 2.23E+06 | 86% |
| Sulfolobus solfataricus | 2.47E+06 | 2.99E+06 | 83% |
| Sulfolobus tokodaii | 2.25E+06 | 2.69E+06 | 83% |
| Thermococcus kodakaraensis KOD1 | 1.91E+06 | 2.09E+06 | 91% |
| Thermofilum pendens Hrk 5 | 1.65E+06 | 1.81E+06 | 91% |
| Thermoplasma acidophilum | 1.37E+06 | 1.56E+06 | 88% |
| Thermoplasma volcanium | 1.35E+06 | 1.58E+06 | 85% |
| Thermoproteus neutrophilus V24Sta | 1.59E+06 | 1.77E+06 | 90% |
| Methanocella arvoryzae MRE50 | 2.71E+06 | 3.18E+06 | 85% |
