# Supplementary material for: An Important Role for Purifying Selection in Archaeal Genome Evolution
Source: mSystems. 2017 Oct 24;2(5):e00112-17. doi: 10.1128/mSystems.00112-17 (PMC5655593; doi:10.1128/mSystems.00112-17)
Supplement: TABLE S4 [file sys005172145st4.docx]

**Table S4** Coding density for bacterial genomes (*n* = 79)

| Genome Name | Coding Base (bp) | Genome Size (bp) | Coding density |
| --- | --- | --- | --- |
| Acidovorax avenae citrulli AAC00-1 | 4.8E+06 | 5.4E+06 | 89.47% |
| Acidovorax sp. JS42 | 4.0E+06 | 4.6E+06 | 88.11% |
| Acinetobacter baumannii ACICU | 3.5E+06 | 4.0E+06 | 87.70% |
| Acinetobacter baylyi ADP1 | 3.2E+06 | 3.6E+06 | 88.80% |
| Actinobacillus pleuropneumoniae L20 | 2.0E+06 | 2.3E+06 | 87.88% |
| Actinobacillus succinogenes 130Z | 2.1E+06 | 2.3E+06 | 89.35% |
| Aeromonas hydrophila hydrophila ATCC 7966 | 4.2E+06 | 4.7E+06 | 88.50% |
| Aeromonas salmonicida salmonicida A449 | 4.2E+06 | 5.0E+06 | 83.02% |
| Anaeromyxobacter dehalogenans 2CP-C | 4.6E+06 | 5.0E+06 | 91.13% |
| Anaeromyxobacter sp. Fw109-5 | 4.8E+06 | 5.3E+06 | 90.09% |
| Arthrobacter aurescens TC1 | 4.6E+06 | 5.2E+06 | 88.05% |
| Arthrobacter sp. FB24 | 4.6E+06 | 5.1E+06 | 89.78% |
| Bacteroides fragilis NCTC 9343 | 4.7E+06 | 5.2E+06 | 88.75% |
| Bacteroides thetaiotaomicron VPI-5482 | 5.7E+06 | 6.3E+06 | 89.89% |
| Bartonella bacilliformis KC583 | 1.2E+06 | 1.4E+06 | 81.53% |
| Bartonella henselae Houston-1 | 1.4E+06 | 1.9E+06 | 73.46% |
| Bifidobacterium adolescentis ATCC 15703 | 1.8E+06 | 2.1E+06 | 87.83% |
| Bifidobacterium longum F8 | 1.7E+06 | 2.4E+06 | 71.71% |
| Bordetella avium 197N | 3.3E+06 | 3.7E+06 | 88.92% |
| Bordetella bronchiseptica RB50 | 4.9E+06 | 5.3E+06 | 92.15% |
| Borrelia afzelii PKo | 1.1E+06 | 1.2E+06 | 87.82% |
| Borrelia burgdorferi B31 | 1.3E+06 | 1.5E+06 | 86.15% |
| Bradyrhizobium japonicum USDA 110 | 7.9E+06 | 9.1E+06 | 86.81% |
| Bradyrhizobium sp. BTAi1 | 7.3E+06 | 8.5E+06 | 85.68% |
| Campylobacter concisus 13826 | 1.8E+06 | 2.1E+06 | 84.72% |
| Campylobacter curvus 525.92 | 1.7E+06 | 2.0E+06 | 87.15% |
| Candidatus Blochmannia floridanus | 5.9E+05 | 7.1E+05 | 84.33% |
| Candidatus Blochmannia pennsylvanicus BPEN | 6.2E+05 | 7.9E+05 | 77.73% |
| Caulobacter crescentus CB15 | 3.6E+06 | 4.0E+06 | 90.61% |
| Caulobacter sp. K31 | 5.2E+06 | 5.9E+06 | 88.07% |
| Chlamydia muridarum MoPn | 9.8E+05 | 1.1E+06 | 90.63% |
| Chlamydia trachomatis A/HAR-13 | 9.6E+05 | 1.1E+06 | 91.08% |
| Enterobacter sakazakii ATCC BAA-894 | 4.0E+06 | 4.5E+06 | 88.05% |
| Enterobacter sp. 638 | 4.1E+06 | 4.7E+06 | 88.31% |
| Francisella philomiragia ATCC 25017 | 1.9E+06 | 2.0E+06 | 91.73% |
| Francisella tularensis tularensis FSC 198 | 1.5E+06 | 1.9E+06 | 79.99% |
| Frankia alni ACN14a | 6.5E+06 | 7.5E+06 | 86.31% |
| Frankia sp. CcI3 | 4.6E+06 | 5.4E+06 | 84.94% |
| Geobacillus kaustophilus HTA426 | 3.1E+06 | 3.6E+06 | 86.33% |
| Geobacillus thermodenitrificans NG80-2 | 3.1E+06 | 3.6E+06 | 85.18% |
| Haemophilus ducreyi 35000HP | 1.5E+06 | 1.7E+06 | 86.73% |
| Haemophilus influenzae R2846 | 1.6E+06 | 1.8E+06 | 88.19% |
| Leuconostoc citreum KM20 | 1.7E+06 | 1.9E+06 | 89.62% |
| Leuconostoc mesenteroides ATCC 8293 | 1.9E+06 | 2.1E+06 | 89.23% |
| Listeria innocua Clip11262 | 2.8E+06 | 3.1E+06 | 89.89% |
| Listeria monocytogenes EGD-e | 2.7E+06 | 2.9E+06 | 90.27% |
| Neisseria gonorrhoeae FA 1090 | 1.7E+06 | 2.2E+06 | 79.21% |
| Neisseria meningitidis 053442 | 1.7E+06 | 2.2E+06 | 80.82% |
| Nitrobacter hamburgensis X14 | 4.0E+06 | 5.0E+06 | 79.63% |
| Nitrobacter winogradskyi Nb-255 | 2.9E+06 | 3.4E+06 | 84.28% |
| Nostoc punctiforme PCC 73102 | 7.0E+06 | 9.1E+06 | 77.43% |
| Nostoc sp. PCC 7120 | 5.9E+06 | 7.2E+06 | 82.50% |
| Polaromonas naphthalenivorans CJ2 | 4.8E+06 | 5.4E+06 | 88.52% |
| Polaromonas sp. JS666 | 5.2E+06 | 5.9E+06 | 87.56% |
| Polynucleobacter necessarius QLW-P1DMWA-1 | 2.0E+06 | 2.2E+06 | 93.14% |
| Polynucleobacter necessarius STIR1 | 1.2E+06 | 1.6E+06 | 78.51% |
| Pseudomonas aeruginosa PAO1 | 5.6E+06 | 6.3E+06 | 89.68% |
| Pseudomonas entomophila L48 | 5.3E+06 | 5.9E+06 | 89.44% |
| Psychrobacter arcticus 273-4 | 2.2E+06 | 2.7E+06 | 81.48% |
| Psychrobacter cryohalolentis K5 | 2.6E+06 | 3.1E+06 | 83.74% |
| Ralstonia eutropha H16 | 6.5E+06 | 7.4E+06 | 87.85% |
| Ralstonia metallidurans CH34 | 6.2E+06 | 6.9E+06 | 89.00% |
| Rhizobium etli CFN 42, DSM 11541 | 5.6E+06 | 6.5E+06 | 85.91% |
| Rhizobium leguminosarum bv. viciae 3841 | 6.7E+06 | 7.8E+06 | 86.81% |
| Rickettsia canadensis McKiel | 8.7E+05 | 1.2E+06 | 75.13% |
| Rickettsia rickettsii Sheila Smith | 9.6E+05 | 1.3E+06 | 76.57% |
| Roseiflexus castenholzii HLO8, DSM 13941 | 4.7E+06 | 5.7E+06 | 82.34% |
| Roseiflexus sp. RS-1 | 4.9E+06 | 5.8E+06 | 85.27% |
| Salinispora arenicola CNS-205 | 5.0E+06 | 5.8E+06 | 86.32% |
| Salinispora tropica CNB-440 | 4.6E+06 | 5.2E+06 | 88.50% |
| Staphylococcus aureus aureus MRSA252 | 2.4E+06 | 2.9E+06 | 82.81% |
| Staphylococcus epidermidis ATCC 12228 | 2.2E+06 | 2.6E+06 | 84.36% |
| Streptomyces avermitilis MA-4680 | 7.9E+06 | 9.1E+06 | 86.43% |
| Streptomyces coelicolor A3(2) | 8.0E+06 | 9.1E+06 | 88.72% |
| Vibrio cholerae MJ-1236 | 3.7E+06 | 4.2E+06 | 87.07% |
| Vibrio fischeri ES114 | 3.8E+06 | 4.3E+06 | 88.03% |
| Yersinia enterocolitica enterocolitica 8081 | 4.0E+06 | 4.7E+06 | 84.35% |
| Yersinia pestis Angola | 3.6E+06 | 4.7E+06 | 77.51% |
